# Hypermetabolism and impaired cerebrovascular reactivity beyond the standard MRI-identified tumor border indicate diffuse glioma extended tissue infiltration

**DOI:** 10.1093/noajnl/vdab048

**Published:** 2021-03-30

**Authors:** Martina Sebök, Christiaan Hendrik Bas van Niftrik, Giovanni Muscas, Athina Pangalu, Katharina Seystahl, Michael Weller, Luca Regli, Jorn Fierstra

**Affiliations:** 1 Department of Neurosurgery, University Hospital Zurich, University of Zurich, Zurich, Switzerland; 2 Clinical Neuroscience Center, University Hospital Zurich, Zurich, Switzerland; 3 Department of Neurosurgery, Careggi University Hospital, Florence, Italy; 4 Department of Neuroradiology, University Hospital Zurich, University of Zurich, Zurich, Switzerland; 5 Department of Neurology, University Hospital Zurich, University of Zurich, Zurich, Switzerland

**Keywords:** BOLD fMRI, cerebrovascular reactivity, glioma, metabolism, PET

## Abstract

**Background:**

Diffuse gliomas exhibit diffuse infiltrative growth, often beyond the magnetic resonance imaging (MRI)-detectable tumor lesion. Within this lesion, hypermetabolism and impaired cerebrovascular reactivity (CVR) are found, but its exact distribution pattern into the peritumoral environment is unknown. Our aim was to better characterize the extent of diffuse glioma tissue infiltration, beyond the visible lesion (ie, beyond the T1-contrast-enhancing lesion and/or T2/FLAIR-defined tumor border), with metabolic positron emission tomography (PET), and functional MRI CVR (blood oxygenation-level-dependent CVR [BOLD-CVR]) mapping.

**Methods:**

From a prospective glioma database, 18 subjects (19 datasets) with diffuse glioma (*n* = 2 with anaplastic astrocytoma, *n* = 10 with anaplastic oligodendroglioma, and *n* = 7 with glioblastoma) underwent a BOLD-CVR and metabolic PET study between February 2016 and September 2019, 7 of them at primary diagnosis and 12 at tumor recurrence. In addition, 19 matched healthy controls underwent an identical BOLD-CVR study. The tumor lesion was defined using high-resolution anatomical MRI. Volumes of interest starting from the tumor lesion outward up to 30 mm were created for a detailed peritumoral PET and BOLD-CVR tissue analysis. Student’s *t* test was used for statistical analysis.

**Results:**

Patients with diffuse glioma exhibit impaired BOLD-CVR 12 mm beyond the tumor lesion (*P* = .02) with normalization of BOLD-CVR values after 24 mm. Metabolic PET shows a difference between the affected and contralateral hemisphere of 6 mm (*P* = .05) with PET values normalization after 12 mm.

**Conclusion:**

We demonstrate hypermetabolism and impaired CVR beyond the standard MRI-defined tumor border, suggesting active tumor infiltration in the peritumoral environment.

**Key Points**

- Hypermetabolism and impaired CVR beyond the standard diffuse glioma border.
- Active infiltration of diffuse glioma in the peritumoral environment.
- Combined hemodynamic and metabolic approach to identify diffuse glioma infiltration.

**Importance of the Study**

Diffuse gliomas exhibit infiltrative growth beyond the standard MRI-detectable tumor lesion. An advanced imaging approach with BOLD-CVR and metabolic PET can potentially better identify the extent of diffuse glioma infiltration. We were able to show that hypermetabolism and impaired cerebrovascular reactivity are present beyond the standard MRI-defined tumor border, suggesting active tumor infiltration in the peritumoral tissue. The hypermetabolism is still significantly present 6 mm beyond the visible glioma lesion, whereas significant BOLD-CVR impairment is seen up to 12 mm. This larger hemodynamic (vascular) impairment could be explained by the recently reported concept that glioma cells have the ability to connect to and integrate into neural circuits in the brain with the formation of neuron–glioma synapses (ie, co-option).

Diffuse gliomas exhibit heterogeneous metabolic and hemodynamic properties, resulting in an erratic growth pattern.^1,2^ This behavior is reflected in neuroimaging challenges, where the tumor infiltration zone usually exceeds the contrast-enhancing lesion on T1-weighted magnetic resonance imaging (MRI), and often does not exactly match the metabolic “hot spots” seen on *O*-(2-[^18^F]fluoroethyl)-l-tyrosine-positron emission tomography (FET-PET).^3–5^ Since accurate delineation of the tumor infiltration zone holds great therapeutic and prognostic value, multimodal neuroimaging approaches are now considered state-of-the-art in order to obtain the most comprehensive tumor imaging characterization.

In this regard, blood oxygenation-level-dependent (BOLD) functional MRI (fMRI) cerebrovascular reactivity (CVR) may provide important complementary hemodynamic information.^6–8^ For noninvasive assessment of brain function to evaluate diffuse glioma surgery in eloquent brain areas, BOLD fMRI is already increasingly used.^9^ BOLD fMRI can be enhanced with a controlled carbon dioxide (CO_2_) stimulus, to allow for detailed CVR mapping of the contrast-enhancing glioma lesion as well as the peritumoral—infiltrative—tumor zone.^8,10,11^ This is of particular interest for the non-contrast-enhancing tumor infiltration zone, where gliomas are believed to cause vascular dysregulation, and neurovascular uncoupling.^6,8,10,11^

Therefore, by acquiring metabolic and hemodynamic neuroimaging data, the extent of diffuse glioma-related tissue disturbances can be investigated. For this study, we aimed at better characterizing diffuse tissue infiltration of diffuse gliomas, beyond the classically defined tumor lesion (ie, beyond the T1 contrast-enhancing lesion and/or T2/FLAIR-defined tumor border), with metabolic PET, and BOLD-CVR mapping.

## Materials and Methods

At the Clinical Neuroscience Center of University Hospital Zurich, patients with IDH-mutant, 1p/19-codeleted anaplastic oligodendroglioma (*n* = 9), IDH-mutant anaplastic astrocytoma (*n* = 2), or IDH-wildtype glioblastoma (*n* = 7) according to the 2016 WHO classification,^1,2^ primary or recurrent, were selected from a prospective database based on following inclusion criteria: patients with age older than 18 years who underwent both a hemodynamic BOLD-CVR imaging and a metabolic imaging with either FET-PET or fluorodeoxyglucose-positron emission tomography (FDG-PET) within the time frame of 6 weeks. Both metabolic PET and BOLD-CVR investigations were performed after the routine contrast-enhanced T1-weighted imaging. The patients underwent BOLD-CVR imaging in additional MR sessions to prevent that CVR results would be affected post-contrast-enhanced.

The study was conducted between February 2016 and September 2019. All the subjects signed an informed consent before participation in this study. The Cantonal Ethics Committee of the Canton Zurich, Switzerland (KEK-ZH-Nr. 2012-0427) approved this study. Some patient data of this cohort were previously reported.^8^

Patients with new neurological symptoms between both scans and subjects with any kind of treatment (surgical, oncological, or radiation) between PET and BOLD-CVR imaging were excluded. One patient with 1p/19-codeleted anaplastic oligodendroglioma underwent the PET and BOLD-CVR mapping 2 times: by a diagnosis of primary glioma and by glioma recurrence.

The diagnosis was confirmed with histopathological analysis in all primary dffuse gliomas, and if available for recurrent gliomas. For the remaining recurrent gliomas (6 of 12), the diagnosis of tumor progression as done by the treating physicians was based on RANO criteria^12^ and confirmed by the interdisciplinary neuro-oncological tumor board.

To determine the normal ranges of whole brain and hemispheric BOLD-CVR, 19 age- and sex-matched healthy subjects were extracted from the same BOLD-CVR database. The criteria for being included within this database were healthy adult subjects with no known neurological symptoms or intracranial pathologies.

The data that support the findings of this study are available from the corresponding author upon reasonable request.

### Image Acquisition and Processing

MRI data were acquired on a 3-T Skyra VD13 (Siemens Healthcare, Erlangen, Germany) with a 32-channel head coil. BOLD fMRI parameters and a 3-dimensional (3D) T1-weighted Magnetization Prepared Rapid Acquisition Gradient Echo (MP RAGE) image were performed. FET-PET scans were performed on an ECAT EXACT HRþ scanner (Siemens Healthcare, Erlangen, Germany).

#### BOLD-CVR determination.

—During the fMRI sequence, the carbon dioxide stimulus was modulated by a computer-controlled gas blender with prospective gas targeting algorithms (RespirAct; Thornhill Research Institute, Toronto, Canada). The RespirAct allows for precise targeting of arterial partial pressure of oxygen and CO_2_.^13^ The controlled hypercapnic stimulus was given to patients during the BOLD-CVR study, as previously published.^14,15^ All the acquired raw BOLD fMRI volumes were transferred to an external computer and preprocessed with SPM 12 (Statistical Parameter Mapping Software; Wellcome Department of Imaging Neuroscience, University College of London, London, UK). The BOLD fMRI volumes were aligned to the T1-weighted MP RAGE image and were smoothed with a Gaussian Kernel of 6 (for more information, see Sebök et al., 2018, Methods).^14^ Next, a voxel-wise temporal shifting for optimal physiological correlation of the BOLD signal and CO_2_ time series was performed. CVR, defined as the percentage BOLD signal change/mmHg CO_2_, was then calculated from the slope of a linear least-square fit of the BOLD signal time course to the CO_2_ time series over the range of the first baseline of 100 s, the step portion of the protocol (80 s) and the second baseline of 100 s on a voxel-by-voxel basis. The extra BOLD fMRI volumes were acquired to allow for the potential temporal shift. The CVR calculations were done as described.^16^

#### Metabolic PET studies: FET-PET and FDG-PET.

—FET-PET scans were performed on an ECAT EXACT HRþ scanner, which acquires 63 contiguous trans-axial planes, simultaneously covering 15.5 cm of the axial field of view. After a 15-min transmission scan (germanium-68 sources), a target dose of 185 MBq of FET was injected intravenously. PET acquisition in 3-dimensional mode was started 30–40 min after injection (128 × 128 matrix). Data were reconstructed by filtered back-projection using a Hann filter after correction for scattering and attenuation.

One patient underwent [^18^F]2-fluoro-2deoxy-d-glucose positron emission tomography with computer tomography (FDG-PET CT) study. The early FDG-PET scans were started 40–45 min after the administration of 8–15 mCi F-18 FDG using a hybrid PET/CT system (Ingenuity, TF PET/CT/Philips, the Netherlands), and the delayed PET/CT scans were performed at 75 min after the early scan. CT data were acquired first and the following parameters were used: tube rotation time, 0.5 s per revolution; 120 kV; 140 mAs; reconstructed slice thickness, 5 mm. After the acquisition of CT data had been completed, the tabletop with the patient automatically advanced into the PET-sensitive field of view and acquisition of PET data was started in 3-dimensional mode with the patient in exactly the same position on the table. Scanning was performed in one-bed position for 3 min. The attenuation correction was automatically completed using corresponding CT data.

### Volumes of Interest Determination

Three-dimensional tumor masks were determined and manually drawn from the current state-of-the-art MRI protocol by a neuroradiologist with more than 20 years of experience (A.P.) using iPlan software (BrainLab AG, Munich, Germany). The tumor borders were drawn on every slice of the T1-weighted contrast enhanced scan where the tumor was visible, or on every slice of FLAIR and/or T2 sequences in case of absent contrast enhancement. For contrast-enhancing tumors, the borders were confirmed and/or adapted regarding the expansion on FLAIR and T2 sequences obtaining a 3-dimensional spherical volume of interest (VOI).

These tumor masks were overlaid on the BOLD-CVR and PET maps to obtain mean intralesional values in those specific regions. Automated spherical 3-dimensional concentric VOIs^17^ of 6 mm width starting from the tumor border outward up to 30 mm (5 VOIs) were created to perform peritumoral tissue analysis. These VOIs were overlaid on the BOLD-CVR and -PET maps to obtain tumor volume, whole brain, gray matter, and white matter mean BOLD-CVR and FET-PET/FDG-PET values. On the contralateral hemisphere in mirror-like anatomical regions (ie, flipped analysis), the same analysis was performed to confirm impaired peritumoral hemodynamic.

### Statistical Analysis

We performed a statistical analysis using SPSS Statistics 26 (IBM Corp., Armonk, NY). Means of normally distributed continuous variables from IDH-mutant WHO grade III and IDH-wildtype IV glioma groups were compared by an independent Student’s 2-tailed *t* test, where *P* < .05 was considered statistically significant. Each VOI was analyzed separately and a comparison between affected and contralateral (ie, flipped) hemisphere was made for BOLD-CVR and FET-PET using paired *t* test. Moreover, paired *t* test was used to identify differences between BOLD-CVR and PET values of VOIs for the whole cohort.

## Results

### Study Population Characteristics

**Figure 1.**
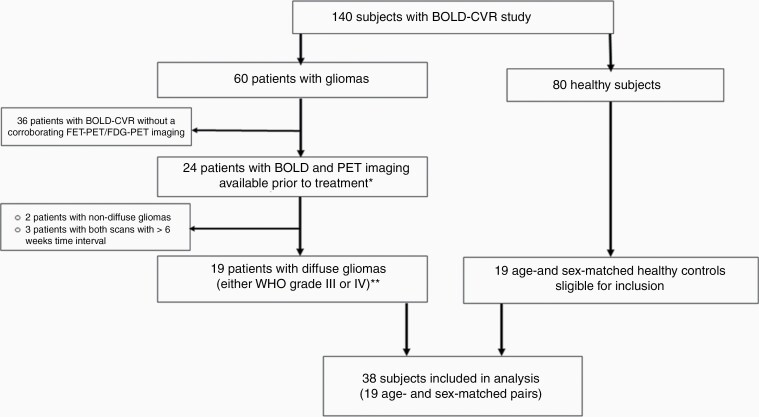
Study flow chart. From the prospective database with 140 subjects who underwent BOLD-CVR study, 60 patients were diagnosed with glioma and 80 were healthy subjects. From 60 glioma patients, 24 patients underwent BOLD and PET imaging prior to treatment (*no treatment [ie, no surgery] done for primary gliomas, no second-line therapy initiated for recurrent glioma) in the time frame of 6 weeks. Two patients with non-diffuse glioma and 3 patients with more than 6 weeks interval between BOLD-CVR and PET scans were excluded from the study. Nineteen subjects with diffuse glioma (either WHO grade III or IV) were eligible for further analysis. **The diagnosis was confirmed with histopathological analysis for all primary diffuse gliomas, and if available for recurrent gliomas. For the remaining recurrent gliomas, diagnosis of progression was done by the treating physicians and based on RANO criteria.^12^ From 80 healthy subjects who underwent the BOLD-CVR study, we extracted 19 age- and sex-matched controls eligible for inclusion. In the final analysis, 38 subjects were included (19 age- and sex-matched pairs).

A flow chart illustrating subject inclusion can be found in Figure 1. Eighteen patients (19 datasets) with diffuse gliomas with mean age 52.4 ± 12.3, 16 males (84%), and 19 age- and sex-matched healthy subjects were included (Table 1). The mean CO_2_ step-change (in mmHg) was similar for diffuse glioma patients and healthy subjects (8.4 ± 1.5 vs 8.3 ± 1.6). A detailed overview of all patient and glioma characteristics can be reviewed in Supplementary Table 1.

**Table 1.** Relevant Characteristics of Glioma Patients and Healthy Controls

	Glioma Cohort (*N* = 19)	Healthy Cohort (*N* = 19)	*P*
Age (mean ± *SD*)	52.4 ± 12.3	52.2 ± 12.0	.96
Sex, male (%)	16 (84.2)	16 (84.2)	n.a.
Mean CVR whole brain	0.15 ± 0.08	0.21 ± 0.08	**.04**
Mean CVR gray matter	0.18 ± 0.08	0.24 ± 0.09	**.03**
Mean CVR white matter	0.12 ± 0.08	0.15 ± 0.05	.24
Mean CVR affected hemisphere^a^	0.16 ± 0.08	0.20 ± 0.08	.06
Mean CVR unaffected hemisphere^a^	0.16 ± 0.08	0.21 ± 0.08	.07

CVR, cerebrovascular reactivity; *N*, number; SD, standard deviation.

^a^For a healthy cohort, the right hemisphere was defined as affected and the left hemisphere as unaffected.

We studied a cohort of IDH-mutant WHO grade III glioma patients (*n* = 2 with anaplastic astrocytoma, *n* = 9 with anaplastic oligodendroglioma) or IDH-wildtype tumors (*n* = 7 with glioblastoma), 7 of them at primary diagnosis and 12 at tumor recurrence. One patient with anaplastic oligodendroglioma underwent the PET and BOLD-CVR mapping 2 times—by a diagnosis of primary glioma and by glioma recurrence—resulting in 19 datasets. Patients with WHO grade III tumors (*n* = 12) were significantly younger. Other baseline characteristics between both groups were comparable.

### BOLD-CVR Findings in Patients With Diffuse Gliomas Versus Healthy Subjects

Patients with diffuse gliomas show significantly impaired whole-brain CVR as compared to the healthy cohort (ie, whole-brain mean CVR value ± standard deviation [SD] for diffuse glioma patients vs healthy subjects: 15 ± 0.08 vs 0.21 ± 0.08; *P* = .04). For gray matter CVR, a distinct difference was also seen (*P* = .03), whereas no difference was found for white matter CVR (Table 1).

### BOLD-CVR Findings in Patients With IDH-Mutant (WHO Grade III) Versus IDH-Wildtype (WHO Grade IV) Glioma

**Figure 2.**
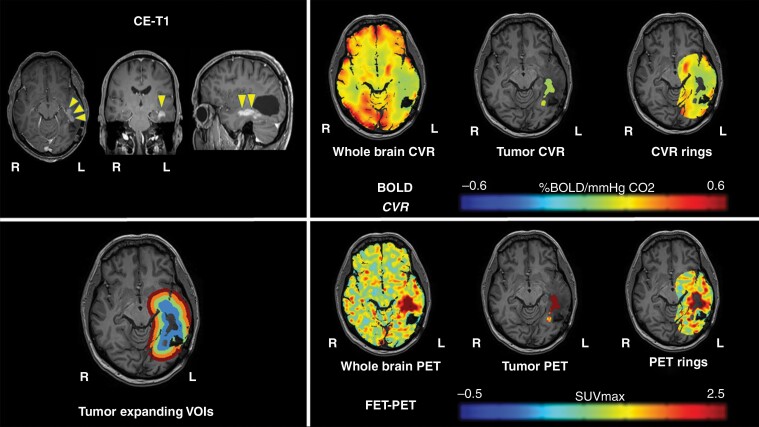
Representative imaging data of one patient with glioblastoma. A 52-year-old male patient with left-sided temporal glioblastoma (WHO grade IV) was diagnosed and macroscopically completely resected in February 2016. In November 2017, the patient presented with recurrent tumor temporal on the left side. He underwent a BOLD-CVR and PET study in a time frame of 6 days and before the microsurgical resection of the recurrent glioma. BOLD-CVR showed impaired CVR clearly beyond the tumor borders as seen in T1-contrast-enhanced images. FET-PET images showed hypermetabolism in the tumor area as well as in the first VOI. The histopathological examination has confirmed the diagnosis of recurrent WHO grade IV glioma.

No differences in whole-brain CVR, gray and white matter CVR were seen between patients with IDH-mutant and IDH-wildtype tumors. Furthermore, tumor volume, CVR, and PET values of the tumor did not show significant differences between both groups. The individual mean CVR values and *P* values can be reviewed in Supplementary Table 2. Representative BOLD-CVR and PET illustrations of one subject with recurrent WHO grade IV glioma are presented in Figure 2.

### BOLD-CVR and PET Finding of Perilesional Brain Tissue: Concentric, Expanding VOI Analysis up to 30 mm From the Visible Glioma Lesion

#### BOLD-CVR and VOI analysis.

—Since we did not find statistically relevant differences in CVR and PET values between patients with IDH-mutant and IDH-wildtype (WHO grade III and IV) glioma, we allowed us to use the entire cohort as one group for the VOI analysis of the peritumoral region. These VOIs consisted of 6-mm-wide volumes that were concentrically expanded up to 30 mm from the visible glioma lesion. Two datasets of the patient with right-sided glioma crossing the midline (patients who underwent BOLD imaging initially by primary tumor diagnosis and by a diagnosis of tumor recurrence) were excluded from this analysis since no reference contralateral hemisphere (ie, flipped analysis) was available for these datasets.

**Figure 3.**
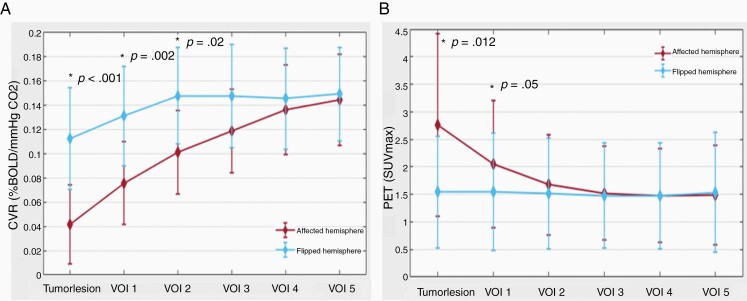
CVR and PET impact of diffuse gliomas on surrounding brain tissue. (A) Line plot of mean CVR values around diffuse glioma. The plot depicts the increase in mean CVR values from the diffuse glioma outward in the surrounding “healthy” brain tissue and also shows lower CVR values in the affected hemisphere. BOLD-CVR values represent the mean values of the 2 hemispheres (red line for affected hemisphere and blue line for flipped hemisphere). (B) Line plot of mean PET values around diffuse glioma. The plot depicts the decrease in mean PET values from the diffuse glioma outward in the surrounding “healthy” brain tissue and also shows higher PET values in the affected hemisphere. PET values represent the mean values of the 2 hemispheres (red line for affected hemisphere and blue line for flipped hemisphere).

Tables 2 and 3 represent the differences in CVR and PET values between the affected hemisphere and flipped (contralateral) hemisphere for the tumor and the concentrically expanded VOIs up to 30 mm of the visible glioma lesion. The hemisphere containing the diffuse glioma (ie, affected hemisphere) exhibited significantly more impaired CVR than the contralateral unaffected hemisphere (mean CVR: 0.03 ± 0.07 vs 0.11 ± 0.08; *P* < .001). Moreover, a difference is seen between the first and second VOI of affected and unaffected hemisphere (0.07 ± 0.06 vs 0.11 ± 0.06; *P* = .002 and 0.10 ± 0.08 vs 0.13 ± 0.04; *P* = .02, respectively). A trend toward normalization of CVR values is observed beginning at an 18 mm distance (ie, VOI 3) from the visible glioma lesion (Table 2 and Figure 3).

**Table 2.** Impact of Supratentorial High-Grade Glioma on Surrounding Brain Tissue: BOLD-CVR Findings

	CVR Affected Hemisphere (*N* = 17^a^)	CVR Contralateral Hemisphere (*N* = 17^a^)	*P*
Supratentorial tumor	0.03 ± 0.07	0.11 ± 0.08	**<.001**
VOI 1 (6 mm)	0.07 ± 0.06	0.11 ± 0.06	**.002**
VOI 2 (12 mm)	0.10 ± 0.08	0.13 ± 0.04	**.02**
VOI 3 (18 mm)	0.12 ± 0.06	0.14 ± 0.06	.20
VOI 4 (24 mm)	0.14 ± 0.09	0.15 ± 0.08	.45
VOI 5 (30 mm)	0.15 ± 0.06	0.15 ± 0.07	.88

CVR, cerebrovascular reactivity; *N*, number; VOI, volume of interest.

^a^Two datasets of patients with right-sided glioma crossing the midline were excluded from calculations.

**Table 3.** Impact of Supratentorial High-Grade Glioma on Surrounding Brain Tissue: PET Findings

	PET Affected Hemisphere (*N* = 17^a^)	PET Contralateral Hemisphere (*N* = 17^a^)	*P*
Supratentorial tumor	2.76 ± 3.32	1.54 ± 2.03	**.012**
VOI 1 (6 mm)	2.25 ± 2.42	1.68 ± 2.28	**.05**
VOI 2 (12 mm)	1.79 ± 1.88	1.59 ± 2.42	.26
VOI 3 (18 mm)	1.61 ± 1.76	1.62 ± 2.18	.67
VOI 4 (24 mm)	1.59 ± 1.64	1.62 ± 2.21	.95
VOI 5 (30 mm)	1.60 ± 1.95	1.67 ± 2.30	.54

*N*, number; PET, positron emission tomography; VOI, volume of interest.

^a^Two datasets of patients with right-sided glioma crossing the midline were excluded from calculations.

Calculating the differences between CVR of the tumor lesion and the VOIs of the affected hemisphere, distinct differences in mean CVR values of the tumor and VOI 1, VOI 1 and VOI 2, as well as VOI 2 and VOI 3 are seen (CVR tumor vs VOI 1: 0.03 ± 0.07 vs 0.07 ± 0.06, *P* < .001; VOI 1 vs VOI 2: 0.07 ± 0.06 vs 0.10 ± 0.08, *P* < .001; VOI 2 vs VOI 3: 0.10 ± 0.08 vs 0.12 ± 0.06, *P* = .008), indicating more severe CVR impairment toward the tumor core. In this regard, no differences between each VOIs are present in the contralateral (“flipped”) hemisphere analysis.

#### Metabolic PET and VOI analysis.

—The standard MRI-defined glioma lesion shows significantly higher PET values compared to the contralateral unaffected hemisphere (2.76 ± 3.32 vs 1.54 ± 2.03; *P* = .012), indicating hypermetabolism. Calculating the difference of the PET values for the VOIs between the affected and contralateral hemispheres, a marked difference is seen only for the first VOI (affected hemisphere vs unaffected hemisphere: 2.25 ± 2.42 vs 1.68 ± 2.28; *P* = .05). Twelve millimeters distant from the hypermetabolic visible glioma lesion (ie, VOI 2), a trend toward normalization of the PET values is seen (Table 3 and Figure 3).

In agreement with the BOLD-CVR findings, the PET VOI analyses show a difference between glioma PET values and the first 3 VOIs (PET tumor vs PET VOI 1: 2.76 ± 3.32 vs 2.25 ± 2.42, *P* = .05; VOI 1 vs VOI 2: 2.25 ± 2.42 vs 1.79 ± 1.88, *P* = .04; VOI 2 vs VOI 3: 1.79 ± 1.88 vs 1.61 ± 1.76, *P* = .05), indicating increasing metabolism toward the tumor core. No significant differences between VOIs are seen for the contralateral (“flipped”) hemisphere for this analysis.

## Discussion

In this study, we demonstrate hypermetabolism and impaired CVR beyond the standard MRI-defined diffuse glioma lesion (ie, beyond the T1 contrast-enhancing lesion and/or T2/FLAIR-defined tumor border), suggesting active tumor infiltration in the peritumoral environment. The hypermetabolism is still significantly present 6 mm beyond the visible glioma lesion, whereas significant BOLD-CVR impairment is seen up to 12 mm. The potential implications, however, point toward a better identification of diffuse glioma tissue infiltration which may improve therapeutic planning and prognostic evaluation in the future. In clinical routine, advanced PET and BOLD-CVR multimodal assessment can be conducted as a single examination when using a PET-MRI system.

### The Meaning of Hemodynamic Impairment and Hypermetabolism Beyond the Standard MRI-Defined Diffuse Glioma Borders

Our results showed that in the immediate vicinity of the tumor environment, there is both hypermetabolism (up to 6 mm) and impaired CVR up to 12 mm beyond the visible diffuse glioma lesion. This larger hemodynamic (vascular) impairment could be explained by the recently reported concept that glioma cells have the ability to connect to and integrate into neural circuits in the brain with the formation of neuron–glioma synapses (ie, co-option). The cancer cells themselves are promoting the neuronal activity that then feeds back to drive the growth of cancer. This could explain why gliomas behave in the way they do—instead of forming a large mass, cancer tends to intertwine throughout the brain.^18^ Moreover, bidirectional interaction between neuron–glioma is present; neuronal activity increases glioma growth,^19^ and gliomas are thought to increase neuronal activity.^18^ Under normal conditions, it is believed that neurons dictate vasodilatation in response to higher levels of CO_2_ resulting in a subsequent increase in cerebral blood flow, ie, physiological neurovascular coupling.^20,21^ Preliminary data in diffuse glioma infer disruption of this coupling mechanism, where perilesional viable neurons cannot enhance regional cerebral blood flow (termed “neurovascular uncoupling”),^6,7,11,22^ a phenomenon that may be more pronounced with increasing tumor volume.^8,23^

This may be related to vascular dysregulation due to tumor neoangiogenesis and altered autoregulation due to the co-option of tumor cells accumulating around the existing vasculature. As a result, the blood vessel walls are destabilized with decreased pericyte coverage, cells thought to be involved in blood flow autoregulation,^20^ leading to the inability of viable neurons to enhance regional cerebral blood flow, something that can be assessed with CVR. In this regard, recent studies reported the presence of peritumoral or even more extended regional impaired CVR, something that was not found for patients harboring low-grade gliomas.^8,24,25^

### Supratentorial Hemodynamic and Metabolic Impairment in Diffuse Glioma

Several previous studies have proven the clinical value of FET-PET imaging to determine the extent of cerebral gliomas for treatment planning, biopsy guidance, detection of tumor recurrence, prognosis, and treatment monitoring.^26–28^ However, our results suggest a potential additional benefit of concomitant BOLD-CVR imaging since a more extended perilesional hemodynamic impairment was found as compared to the hypermetabolism detected with PET.

As for our cohort, diffuse glioma represents a heterogeneous disease. In particular, recurrent gliomas also exhibit radionecrosis and/or gliosis tissue with hyalinized vessels,^29,30^ which impacts CVR and metabolism. Wiestler et al.^31^ presented a multiparametric MRI-based differentiation of WHO grade II/III glioma and glioblastoma with BOLD MRI measurement of oxygen extraction fraction (rOEF) in glioma patients. Among 37 patients with primary gliomas based on multiple MRI modalities (rOEF and perfusion imaging), the WHO grade was correctly predicted in 91.8% of patients. The study suggested that multimodal noninvasive MRI information reflects the underlying tumor biology and showed the importance of the development of the field of radiogenomics. In this regard, the suspected ability of radiological multimodal diffuse glioma characterization is a promising development for both glioma diagnosis and treatment. However, we have to be aware that this largely depends on the quality of the data (ie, better tumor depiction and multimodal imaging characteristics), which may also improve radiogenomics and big data analysis.

### Limitations

In this preliminary study, we have only included 19 datasets of 18 patients with diffuse glioma, numbers that are similar to other studies investigating metabolic and hemodynamic alterations in gliomas.^31–33^ Therefore, the results should be interpreted with caution. Second, this was a retrospective analysis of prospectively collected data. The third important limitation of our study is that we included a mixed cohort of diffuse glioma patients with WHO grade III and grade IV gliomas as well as primary and recurrent gliomas. Recurrent gliomas are often not a homogenous tumorous tissue that can affect our data. Moreover, for 6 of 12 patients with recurrent tumors, no histological tissue at tumor recurrence is available and diagnosis of tumor progression as done by the treating physicians was based on RANO criteria,^12^ which might not fully exclude that areas of radionecrosis contribute to the radiological properties of the tumor. Furthermore, the included patients underwent a metabolic imaging with either FET-PET or FDG-PET. A well-known physiological limitation of the BOLD-CVR technique—imaging resolution and smoothing technique—could affect the BOLD-CVR values in the first VOI around the anatomically defined tumor lesion. To overcome this limitation, for our future projects, we are planning to determine BOLD-CVR values around the PET-determined tumor lesion, meaning in the peritumoral tissue with PET enhancement but without contrast agent enhancement. Since gliomas are very infiltrative tumors using the contralateral hemisphere for flipped VOI analysis, an occult tumor tissue as a confounder could be introduced. The analysis using the infratentorial normalization would allow for the normalization of each ring and a more optimal intersubject comparison. However, our aim was to compare the tissue in different rings supratentorial to the same tissue contralateral (using an intrasubject analysis). This confounder would result in a type 2 error (false negative).

## Conclusions

In patients with diffuse glioma, we demonstrate hypermetabolism and impaired CVR beyond the MRI-detectable tumor border, suggesting active tumor infiltration in the peritumoral microenvironment. This advanced multimodal imaging approach can potentially better identify the extent of erratic infiltration of diffuse gliomas; however, its merit needs to be confirmed prospectively.

## Supplementary Material

vdab048_suppl_Supplementary_Materials

## Notes

**Authorship Statement.** M.S., C.H.B.v.N., M.W., L.R., and J.F. contributed to the conception and design of the study. M.S., C.H.B.v.N., G.M., A.P., and K.S. contributed to the acquisition and analysis of data. M.S., C.H.B.v.N., L.R., and J.F. drafted a significant portion of the manuscript or figures.

## References

[1] Louis DN , Perry A, Reifenberger G, et al. The 2016 World Health Organization Classification of tumors of the central nervous system: a summary. Acta Neuropathol. 2016;131(6):803–820. 27157931 10.1007/s00401-016-1545-1

[2] Weller M , van den Bent M, Preusser M, et al. EANO guidelines on the diagnosis and treatment of diffuse gliomas of adulthood. Nat Rev Clin Oncol. 2020;18(3):170–186. 10.1038/s41571-020-00447-z PMC7904519 33293629

[3] Kunz M , Thon N, Eigenbrod S, et al. Hot spots in dynamic (18)FET-PET delineate malignant tumor parts within suspected WHO grade II gliomas. Neuro Oncol. 2011;13(3):307–316. 21292686 10.1093/neuonc/noq196 PMC3064604

[4] Da Silva NA , Lohmann P, Fairney J, et al. Hybrid MR-PET of brain tumours using amino acid PET and chemical exchange saturation transfer MRI. Eur J Nucl Med Mol Imaging. 2018;45(6):1031–1040. 29478081 10.1007/s00259-018-3940-4

[5] Marner L , Henriksen OM, Lundemann M, Larsen VA, Law I. Clinical PET/MRI in neurooncology: opportunities and challenges from a single-institution perspective. Clin Transl Imaging. 2017;5(2):135–149. 28936429 10.1007/s40336-016-0213-8 PMC5581366

[6] Pillai JJ , Mikulis DJ. Cerebrovascular reactivity mapping: an evolving standard for clinical functional imaging. AJNR Am J Neuroradiol. 2015;36(1):7–13. 24788129 10.3174/ajnr.A3941 PMC7965914

[7] Zaca D , Hua J, Pillai JJ. Cerebrovascular reactivity mapping for brain tumor presurgical planning. World J Clin Oncol. 2011;2(7):289–298. 21773079 10.5306/wjco.v2.i7.289 PMC3139032

[8] Fierstra J , van Niftrik C, Piccirelli M, et al. Diffuse gliomas exhibit whole brain impaired cerebrovascular reactivity. Magn Reson Imaging. 2018;45:78–83. 28986176 10.1016/j.mri.2017.09.017

[9] Pillai JJ , Zacà D. Comparison of BOLD cerebrovascular reactivity mapping and DSC MR perfusion imaging for prediction of neurovascular uncoupling potential in brain tumors. Technol Cancer Res Treat. 2012;11(4):361–374. 22376130 10.7785/tcrt.2012.500284

[10] Fierstra J , van Niftrik B, Piccirelli M, et al. Altered intraoperative cerebrovascular reactivity in brain areas of high-grade glioma recurrence. Magn Reson Imaging. 2016;34(6):803–808. 26968146 10.1016/j.mri.2016.02.003

[11] Holodny AI , Schulder M, Liu WC, Maldjian JA, Kalnin AJ. Decreased BOLD functional MR activation of the motor and sensory cortices adjacent to a glioblastoma multiforme: implications for image-guided neurosurgery. AJNR Am J Neuroradiol. 1999;20(4):609–612. 10319970 PMC7056038

[12] Wen PY , Macdonald DR, Reardon DA, et al. Updated response assessment criteria for high-grade gliomas: response assessment in neuro-oncology working group. J Clin Oncol. 2010;28(11):1963–1972. 20231676 10.1200/JCO.2009.26.3541

[13] Slessarev M , Han J, Mardimae A, et al. Prospective targeting and control of end-tidal CO_2_ and O_2_ concentrations. J Physiol. 2007;581(Pt 3):1207–1219. 17446225 10.1113/jphysiol.2007.129395 PMC2170842

[14] Sebok M , van Niftrik CHB, Piccirelli M, et al. BOLD cerebrovascular reactivity as a novel marker for crossed cerebellar diaschisis. Neurology. 2018;91(14):e1328–e1337. 10.1212/WNL.0000000000006287 30185447

[15] van Niftrik CHB , Piccirelli M, Bozinov O, et al. Impact of baseline CO_2_ on Blood-Oxygenation-Level-Dependent MRI measurements of cerebrovascular reactivity and task-evoked signal activation. Magn Reson Imaging. 2018;49:123–130. 29447850 10.1016/j.mri.2018.02.002

[16] van Niftrik CHB , Piccirelli M, Bozinov O, et al. Iterative analysis of cerebrovascular reactivity dynamic response by temporal decomposition. Brain Behav. 2017:e00705. 28948064 10.1002/brb3.705 PMC5607533

[17] Fierstra J , Conklin J, Krings T, et al. Impaired peri-nidal cerebrovascular reserve in seizure patients with brain arteriovenous malformations. Brain. 2011;134(Pt 1):100–109. 21109501 10.1093/brain/awq286

[18] Venkatesh HS , Morishita W, Geraghty AC, et al. Electrical and synaptic integration of glioma into neural circuits. Nature. 2019;573(7775):539–545. 31534222 10.1038/s41586-019-1563-y PMC7038898

[19] Venkatesh HS , Johung TB, Caretti V, et al. Neuronal activity promotes glioma growth through neuroligin-3 secretion. Cell. 2015;161(4):803–816. 25913192 10.1016/j.cell.2015.04.012 PMC4447122

[20] Attwell D , Buchan AM, Charpak S, Lauritzen M, Macvicar BA, Newman EA. Glial and neuronal control of brain blood flow. Nature. 2010;468(7321):232–243. 21068832 10.1038/nature09613 PMC3206737

[21] Heeger DJ , Huk AC, Geisler WS, Albrecht DG. Spikes versus BOLD: what does neuroimaging tell us about neuronal activity? Nat Neurosci. 2000;3(7):631–633. 10862687 10.1038/76572

[22] Hou BL , Bradbury M, Peck KK, Petrovich NM, Gutin PH, Holodny AI. Effect of brain tumor neovasculature defined by rCBV on BOLD fMRI activation volume in the primary motor cortex. Neuroimage. 2006;32(2):489–497. 16806983 10.1016/j.neuroimage.2006.04.188

[23] Wang Q , Zhang H, Zhang J, et al. The diagnostic performance of magnetic resonance spectroscopy in differentiating high-from low-grade gliomas: a systematic review and meta-analysis. Eur Radiol. 2015;26(8):2670–2684. 10.1007/s00330-015-4046-z 26471274

[24] Hsu YY , Chang CN, Jung SM, et al. Blood oxygenation level-dependent MRI of cerebral gliomas during breath holding. J Magn Reson Imaging. 2004;19(2):160–167. 14745748 10.1002/jmri.10447

[25] Ludemann L , Forschler A, Grieger W, Zimmer C. BOLD signal in the motor cortex shows a correlation with the blood volume of brain tumors. J Magn Reson Imaging. 2006;23(4):435–443. 16506145 10.1002/jmri.20530

[26] Filss CP , Galldiks N, Stoffels G, et al. Comparison of 18F-FET PET and perfusion-weighted MR imaging: a PET/MR imaging hybrid study in patients with brain tumors. J Nucl Med. 2014;55(4):540–545. 24578243 10.2967/jnumed.113.129007

[27] Pauleit D , Floeth F, Hamacher K, et al. *O*-(2-[^18^F]fluoroethyl)-l-tyrosine PET combined with MRI improves the diagnostic assessment of cerebral gliomas. Brain. 2005;128(Pt 3):678–687. 15689365 10.1093/brain/awh399

[28] Popperl G , Gotz C, Rachinger W, Gildehaus FJ, Tonn JC, Tatsch K. Value of *O*-(2-[^18^F]fluoroethyl)-l-tyrosine PET for the diagnosis of recurrent glioma. Eur J Nucl Med Mol Imaging. 2004;31(11):1464–1470. 15248032 10.1007/s00259-004-1590-1

[29] Woodworth GF , Garzon-Muvdi T, Ye X, Blakeley JO, Weingart JD, Burger PC. Histopathological correlates with survival in reoperated glioblastomas. J Neuro-Oncol. 2013;113(3):485–493. 10.1007/s11060-013-1141-3 PMC3994532 23666202

[30] De Wit MC , de Bruin HG, Eijkenboom W, Sillevis Smitt PA, van den Bent MJ. Immediate post-radiotherapy changes in malignant glioma can mimic tumor progression. Neurology. 2004;63(3):535–537. 15304589 10.1212/01.wnl.0000133398.11870.9a

[31] Wiestler B , Kluge A, Lukas M, et al. Multiparametric MRI-based differentiation of WHO grade II/III glioma and WHO grade IV glioblastoma. Sci Rep. 2016;6:35142. 27739434 10.1038/srep35142 PMC5064384

[32] Tien RD , Ashdown BC. Crossed cerebellar diaschisis and crossed cerebellar atrophy: correlation of MR findings, clinical symptoms, and supratentorial diseases in 26 patients. AJR Am J Roentgenol. 1992;158(5):1155–1159. 1566683 10.2214/ajr.158.5.1566683

[33] Patay Z , Parra C, Hawk H, et al. Quantitative longitudinal evaluation of diaschisis-related cerebellar perfusion and diffusion parameters in patients with supratentorial hemispheric high-grade gliomas after surgery. Cerebellum (London, England). 2014;13(5):580–587. 10.1007/s12311-014-0575-2 24917518

