# Supplementary material for: Hypermetabolism and impaired cerebrovascular reactivity beyond the standard MRI-identified tumor border indicate diffuse glioma extended tissue infiltration
Source: Neurooncol Adv. 2021 Mar 30;3(1):vdab048. doi: 10.1093/noajnl/vdab048 (PMC8156976; doi:10.1093/noajnl/vdab048)
Supplement: vdab048_suppl_Supplementary_Materials [file vdab048_suppl_supplementary_materials.docx]

**Supplementary tables**

Supplementary Table 1: Subject characteristics

| **Case** | **Age**  **(years) #** | **Sex** | **Tumor**  **location and contrast-enhancement** | **Histology and**  **WHO-grade** | | **IDH and**  **MGMT**  **status** | | **Dexamethasone therapy at the time point of imaging** | | | | | | **Primary/**  **Recurrence** | **Time between PET and BOLD (days)** | | |  |
| --- | --- | --- | --- | --- | --- | --- | --- | --- | --- | --- | --- | --- | --- | --- | --- | --- | --- | --- |
| 1 | 39 | M | Left frontal,  non-CE tumor | Anaplastic  astrocytoma  (WHO III) | | IDH pos.  MGMT pos. | | No | | | Recurrence | | | | 35 | | |  |
| 2 | 32 | F | Right temporal,  CE tumor | Anaplastic astrocytoma (WHO III) | | IDH pos.  MGMT neg. | | No | | | Recurrence | | | | 11 | | |  |
| 3 | 63 | M | Left temporal,  CE tumor | Glioblastoma  (WHO IV) | | IDH wildtype  MGMT neg. | | Yes | | | Primary | | | | 23 | | |  |
| 4 | 37 | M | Right frontal,  non-CE tumor | Anaplastic oligodendroglioma  (WHO III) | | IDH pos.  MGMT neg. | | No | | | Primary | | | | 13 | | |  |
| 5 | 45 | M | Right temporo-parietal,  CE tumor | Anaplastic oligodendroglioma  (WHO III) | | IDH pos.  MGMT neg. | | | No | | | | Recurrence | | 33 | | |  |
| 6 | 70 | F | Right frontal,  CE tumor | Glioblastoma  (WHO IV) | | IDH wildtype  MGMT neg. | | | Yes | | | Primary | | | 17 | | |  |
| 7 | 61 | F | Left parietal,  CE tumor | Glioblastoma  (WHO IV) | | IDH wildtype  MGMT neg. | | | No | | | Recurrence | | | 10 | | |  |
| 8 | 52 | M | Left frontal,  non-CE tumor | Anaplastic oligodendroglioma  (WHO III) | | IDH pos.  MGMT neg. | | | No | | | Primary | | | 2 | | |  |
| 9 | 52 | M | Left frontal,  non-CE tumor | Anaplastic oligodendroglioma  (WHO III) | | IDH pos.  MGMT pos. | | | No | | | Recurrence | | | 35 | | |  |
| 10 | 54 | M | Left temporo-occipital,  CE tumor | Glioblastoma  (WHO IV) | | IDH wildtype  MGMT pos. | | | No | | | Recurrence | | | 6 | | |  |
| 11 | 60 | M | Right frontal,  CE tumor | Anaplastic oligodendroglioma  (WHO III) | | | IDH pos.  MGMT neg. | | No | | Recurrence | | | | 17 | | |  |
| 12 | 59 | M | Right frontal,  CE tumor | Anaplastic oligodendroglioma  (WHO III) | | | IDH pos.  MGMT neg. | | No | | Recurrence | | | | | 24 | | |
| 13 | 28 | M | Right frontal,  CE tumor | Anaplastic oligodendroglioma  (WHO III) | | | IDH pos.  MGMT neg. | | No | | Recurrence | | | | | 31 | | |
| 14 | 49 | M | Right frontal,  CE tumor | Glioblastoma  (WHO IV) | IDH wildtype  MGMT neg. | | | | Yes | | Recurrence | | | | | 12 | | |
| 15 | 48 | M | Left temporal,  non-CE tumor | Anaplastic oligodendroglioma  (WHO III) | IDH pos.  MGMT neg. | | | | No | | Recurrence | | | | | 41 | | |
| 16 | 72 | M | Left multifocal (basal ganglia, hippocampus, mesencephalon),  CE tumor | Glioblastoma  (WHO IV) | IDH wildtype  MGMT pos. | | | | Yes | | | Primary | | | | 11 | | |
| 17 | 68 | M | Right temporal,  CE tumor | Glioblastoma  (WHO IV) | IDH wildtype  MGMT neg. | | | | Yes | | | Primary | | | | 24 | | |
| 18***** | 51 | M | Right frontal,  CE tumor | Anaplastic  oligodendroglioma  (WHO III) | IDH pos.  MGMT neg. | | | | No | | | Primary | | | | 0 | | |
| 19***** | 53 | M | Right frontal,  CE tumor | Anaplastic oligodendroglioma  (WHO III) | IDH pos.  MGMT neg. | | | | No | Recurrence | | | | | | 3 |  |  |
| *this patient underwent the protocol two times (initial and by tumor recurrence)  # age at primary diagnosis/age at recurrence  CE: contrast-enhancing, IDH: isocitrate dehydrogenase, MGMT: O^6^-methylguanine-DNA methyl-transferase, neg: negative, pos: positive, WHO: World Health Organization | | | | | | | | | | | | | | | | | |  |

Supplementary Table 2: Supratentorial BOLD-CVR and PET characteristics: IDH mutant vs. IDH wildtype gliomas

| **Functional measurement**  **(mean ± standard deviation)** | **Total cohort**  **(n=19)** | **IDH mutant**  **(WHO grade III;**  **n=12)** | **IDH wildtype (WHO grade IV;**  **n=7)** | **p- value** |
| --- | --- | --- | --- | --- |
| Mean BOLD-CVR whole brain | 0.16 ± 0.08 | 0.17 ± 0.08 | 0.13 ± 0.07 | 0.30 |
| Mean CVR grey matter | 0.18 ± 0.08 | 0.19 ± 0.09 | 0.15 ± 0.07 | 0.23 |
| Mean CVR white matter | 0.12 ± 0.08 | 0.13 ± 0.08 | 0.10 ± 0.08 | 0.45 |
| Mean BOLD-CVR  affected hemisphere | 0.23 ± 0.31 | 0.17 ± 0.08 | 0.12 ± 0.07 | 0.18 |
| Mean BOLD-CVR  unaffected hemisphere | 0.16 ± 0.08 | 0.13 ± 0.20 | 0.13 ± 0.07 | 0.98 |
| Tumor volume (mm^3^) | 21.66 ± 39.38 | 32.58 ± 49.44 | 8.64 ± 7.09 | 0.26 |
| CVR tumor | 0.04 ± 0.07 | 0.03 ± 0.06 | 0.04 ± 0.09 | 0.87 |
| PET tumor | 2.75 ± 3.14 | 2.15 ± 2.03 | 3.78 ± 4.47 | 0.29 |
| CVR = cerebrovascular reactivity, defined as percentage BOLD signal change per mmHg CO_2_, n = number; | | | | |
